# The Influence of rhBMP-7 Associated with Nanometric Hydroxyapatite Coatings Titanium Implant on the Osseointegration: A Pre-Clinical Study

**DOI:** 10.3390/polym14194030

**Published:** 2022-09-26

**Authors:** Rafael Silva Bonato, Gustavo Vicentis de Oliveira Fernandes, Monica Diuana Calasans-Maia, Alexandre Mello, Alexandre Malta Rossi, Ana Claudia Oliveira Carreira, Mari Cleide Sogayar, José Mauro Granjeiro

**Affiliations:** 1 Faculty of Dentistry, Fluminense Federal University, Niteroi 24020-140, RJ, Brazil; 2 Periodontics and Oral Medicine Department, University of Michigan School of Dentistry, Ann Arbor, MI 48109, USA; 3 Brazilian Center for Physics Research, CBPF, Rio de Janeiro, 22290-180, RJ, Brazil; 4 Center for Natural and Human Sciences, Federal University of ABC, Santo Andre 09210-170, SP, Brazil; 5 Cell and Molecular Therapy Center, School of Medicine, Division to Support Research, Training, and Innovation (DATAPEPI), University of São Paulo, São Paulo 05508-060, SP, Brazil; 6 Biochemistry Department, Chemistry Institute, University of São Paulo (USP), Sao Paulo 05508-060, SP, Brazil; 7 National Institute of Metrology, Quality and Technology (InMetro), Duque de Caxias, 25250-020, RJ, Brazil

**Keywords:** bone morphogenetic proteins, pre-clinical study, coated materials, dental implants

## Abstract

**Background**: Bioceramic nanometer coatings have been regarded as potential substitutes for plasma-sprayed hydroxyapatite coatings, and the association with bone morphogenetic protein (BMP) is an attempt to achieve faster osseointegration to hasten oral rehabilitation. **Objective**: This study aimed to investigate the effect of recombinant human bone morphogenetic protein-7 (rhBMP-7) on the osseointegration of titanium implants coated with a thin film surface of hydroxyapatite (HA). **Methods**: Two implants (*n* = 24) were placed in each white New Zealand rabbits’ femur (*n* = 6). Implants were placed in the right femur after standard instrumentation (A and B) and in the left femur after an over-instrumentation (C and D), preventing bone-implant contact. The distal implants were installed associated with rhBMP-7 (groups B [regular instrumentation] and D [over-instrumentation]) and, also, in the absence of without BMP (control groups A [regular instrumentation] and C [over-instrumentation]). After 4 weeks, the animals were euthanized. The bone blocks containing the implants were embedded in methyl methacrylate and sectioned parallel to the long axis of the implant, which were analyzed by image segmentation. The data were analyzed using a nonparametric statistical method. **Results**: We observed that Group A had a mean bone formation of 35.6% compared to Group B, which had 48.6% (*p* > 0.05). Moreover, this group showed 28.3% of connective tissue compared to Group A, with 39.3%. In the over-instrumented groups, rhBMP-7 (Group D) showed an enhanced and significant increase in bone formation when compared with the group without rhBMP-7 (Group C). **Conclusion**: We concluded that the association of rhBMP-7 to thin nanostructure HA-coated implants promoted greater new bone area than the same implants in the absence of rhBMP-7, mainly in cases of over-instrumented implant sites.

## 1. Introduction

Dental implants have recently been widely used, with high success rates been achieved [1]. However, longitudinal studies and histological data have shown that success for endosseous implants varies with the patient’s systemic conditions and local bone quality and quantity [2]. Moreover, the implant must be inserted with a low-trauma surgical technique and placed with sufficient primary stability [3].

The osseointegration phenomenon may take months after implant placement in oral rehabilitation with dental implants. However, reducing the treatment period may be interesting for clinicians and patients [4]. In order to increase the bone-to-implant contact (BIC) at the early response stage, several implant design alterations have been attempted [5]. Following mechanical-chemical treatments [6,7,8,9,10], calcium phosphate implant surface coatings [11,12,13,14], and the association with peptide growth factors (bone morphogenetic proteins) have been investigated, aiming at a faster osseointegration process [15,16,17,18].

Since the clinical success of oral implants is related to their osseointegration, geometry and surface topography play a crucial role in the short- and long-term success of dental implants [5]. Modifying surface topography and chemistry represents quite a challenge, and one strategy has been to use plasma-sprayed HA coatings on implants. Most commercially available bioceramic coated implants are prepared as 20 to 50 µm-thick plasma-sprayed hydroxyapatite coatings [14]. However, failures due to the weak connection among bulk metal, oxides, and bioceramic, associated with the difficulty in reproducing a uniform coating composition and crystallinity, render its manufacture a great challenge. The advent of nanotechnology propelled the development of 100 nm HA-coated implants [19,20], intending to overcome problems related to the previous methods used for surface treatments and generate better results [21].

The general role of bone morphogenetic proteins (BMPs) in bone formation during the development and repair of fractures has been well studied, and its osteoinductive potential has been confirmed both by its deployment in ectopic sites and bone defects of critical size [17,22,23]. Clinical studies and meta-analysis also demonstrated the effectiveness of BMPs in the induction of bone repair in lesions, such as pseudoarthrosis [24], arthrodesis [25], and alveolar bone [26]. Moreover, a wide body of evidence suggests a positive effect of BMP coating titanium surface on the implant osseointegration [27]. Moreover, two BMPs, namely BMP-2 and BMP-7, have received more attention concerning bone formation, both of which promoted bone healing, with no significant differences being found in their healing efficacy [28].

Therefore, the present study evaluated the influence of recombinant human bone morphogenetic protein-7 (rhBMP-7) on the volume density of new bone formation, onto a Ca- and P-based 100 nm thickness bioceramic deposition on a plateau root from a Ti-6Al-4V implants in a rabbit model, comparing regular and over-instrumented perforations. The positive hypothesis is that a higher bone volume density may be observed for the thin-coated implants associated with rhBMP-7.

## 2. Materials and Methods

### 2.1. Implant Surface and Characterization

Titanium (Ti) implants of 3.25 mm × 10.0 mm (*n* = 24) were provided by SIN Implant System, Sao Paulo, Brazil). The surface treatment with hydroxyapatite-thin film (100 nm thick) was completed at the Brazilian Center for Physics Research (CBPF/RJ).

Stoichiometric HA powder (Ca/P = 1.67) was synthesized by dropwise addition of calcium nitrate and ammonium phosphate solutions. The sputtering targets with a diameter of 25 mm were prepared by uniaxially pressing HA under 30 MPa, followed by sintering at 1100 °C. Titanium plates of 1 cm × 1 cm long (prepared precisely by the same process as the commercial titanium implants) and commercial titanium implants were ultrasonically cleaned with 10% hydrofluoric acid and acetone before the oxidization layer. Hydroxyapatite coatings were prepared by right-angle magnetron sputtering (RAMS) at room temperature and with no further heat treatment. Nanometric HA coatings with different thickness levels was grown onto the Ti substrates (plates and dental implants) with a deposition rate of 5.2 nm/minutes and an RF frequency of 13.56 MHz. The partial pressure of Ar and O_2_ in the deposition chamber were 5 and 1 mTorr, respectively.

**Figure 1:**
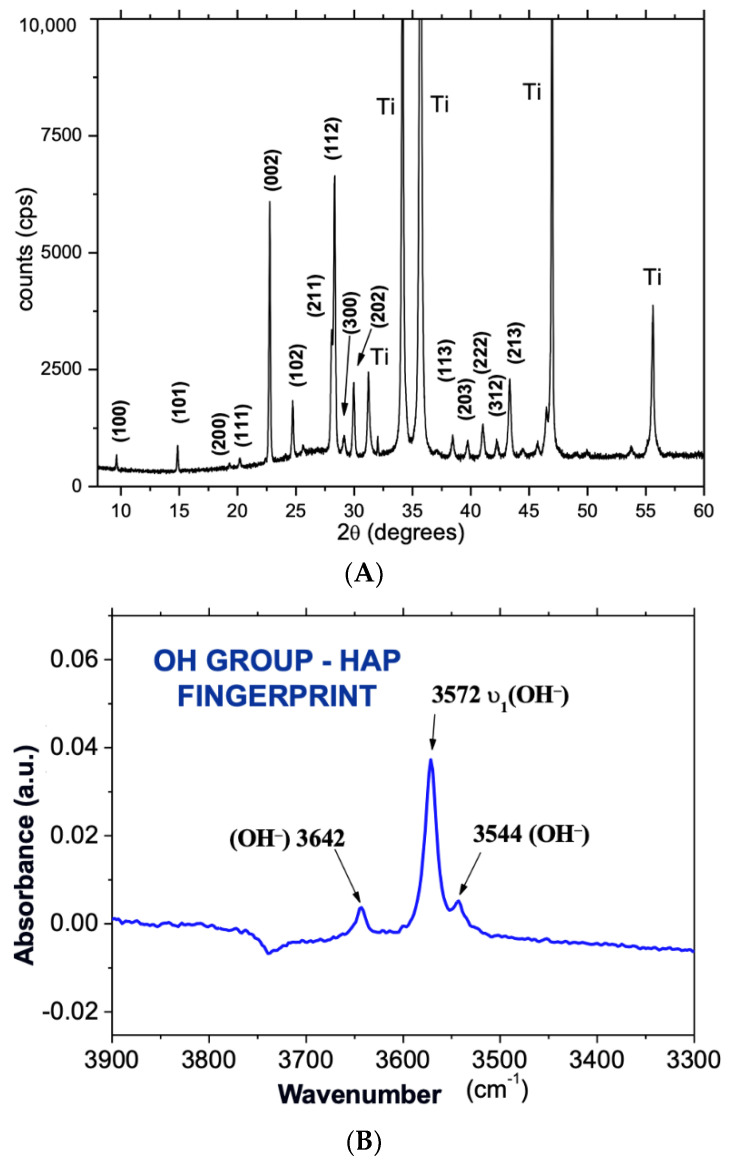
(**A**) XRD of the implant after the surface coating. The diffraction peaks show highly crystalline hydroxyapatite with crystallites preferentially oriented along the axis c; (**B**) OH vibration bands in the FTIR spectrum of as-sputtered HA coatings for 180 min and 120 W RF power sputtering time.

After deposition, the samples were characterized with XRD (Ti plate), FTIR, and SEM (dental implant). The XRD technique was used to identify the phase composition over the deposited thin coating layer of the HA sputtered coatings (Figure 1A) [19,20]. The surface plate samples were characterized by XRD, and grazing-incidence X-ray diffraction (GIXRD) performed with synchrotron radiation, operating at an energy of 9000 eV, wavelength of 0.137 nm, fixed incident angle theta = 0.5° and 1°, and two-theta in the range of 9° to 50° at a rate of 0.04°/point/second.

**Figure 2:**
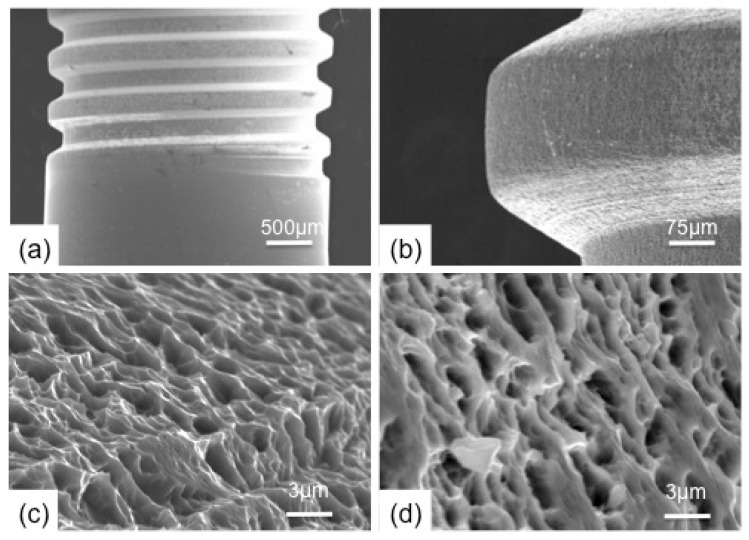
Characterization of implant surfaces by SEM. (**a**,**c**) implant without surface treatment; (**b**,**d**) implantation after coating with a thin film of hydroxyapatite (300 nm).

These methods provided a higher X-ray photon density, leading to a better pick resolution than those obtained with commercial machines. The XRD analysis was performed at the Brazilian Synchrotron Light National Laboratory (LNLS, Campinas/SP, Brazil). In addition, the HA vibrational bands and mainly the OH band (Figure 1B) for the as-sputtered coatings were obtained by Fourier Transformed Infrared Attenuated Total Reflectance Microscopy (FTIRM-ATR) using a Shimadzu IR-Prestige-21/AIM-880 (Shimadzu Corporation, Kyoto, Japan) operating from 700 to 4000 cm^−1^. Before and after HA deposition, the titanium implant surface morphology was characterized by SEM using a Jeol JSM-5800 (Akishima, Tokyo, Japan) operating at 20 kV (Figure 2).

The rhBMP-7 was provided by the Cell and Molecular Therapy Center (www.usp.br/nucel, accessed on 20 August 2022), Department of Biochemistry, Chemistry Institute, University of São Paulo (USP, São Paulo, Brazil), at a concentration of 43 mg/mL, diluted to 2.0 mg/mL, and stored in a sterile microtube at 4 °C until use [29].

### 2.2. In Vivo Experiment

This study was developed according to the standards recommended by the National Council for the Control of Animal Experimentation (CONCEA, Brazil) and approved by the Ethics Committee for Animal Research (CEUA) of the Fluminense Federal University (UFF, No. 005/07). Six White New Zealand rabbits, weighing between 2.5 kg and 3.0 kg, were enrolled. They were anesthetized with general anesthesia, with the administration of 20 mg/kg ketamine (Clortamina^®^) and 1 mg/kg xylazine (Rompun^®^) intramuscularly, maintained with isoflurane (Isoran^®^) at 1% inhalatory, and local infiltration with prilocaine hydrochloride 3% with felypressin (Prilonest^®^).

Following the trichotomy and disinfection of both femurs, a 4-cm incision was made in the lining epithelium of the animal leg. The soft tissue and periosteum were elevated and reflected, exposing the femur bone surface. Two perforations were applied to each femur; the proximal ones being filled with implants without rhBMP-7, and the distal ones with implants embedded in rhBMP-7. Instrumentation in the right femur was performed until the 3.0 mm∅ drill for standard implant installation. In the left femur, an over-instrumentation was carried out until 4.0 mm∅ drill, preventing bone-implant contact throughout the length of the surgical defect. 

The implants were immersed for 15 min in rhBMP-7 (2.0 mg/mL) before placement. The flap was repositioned, and the skin was closed with interrupted #5.0 nylon sutures (Johnson & Johnson, Sao Paulo, Brazil). New disinfection with Chlorhexidine 2% was carried out to prevent further contamination. After the procedure, the animals received 4mg/Kg of Tramadol hydrochloride (Tramal^®^), Meloxicam 0.3 mg/kg (Maxicam^®^), and Enhanced Pentabiotic for veterinary use (Fort Dodge^®^) 0.1 mg/kg, intramuscularly and in a single dose.

Following the study period (four weeks after surgery), the rabbits were euthanized with an overdose of anesthetic agents. The two fragments of each femur with the implants were collected and fixed in 70% alcohol prior to dehydration in successive alcohol solutions and then impregnated and embedded in methyl-methacrylate. The specimens sections of 30–50 µm thickness were not stained and not pasted to the slide.

### 2.3. Histomorphometric Analysis

Histomorphometric analysis was performed on the digital images obtained, which were captured through a digital camera (Evolution^®^ MP Color, 5.0 megapixels; Media Cybernetics, Silver Spring, MD, USA), coupled to a microscope of polarized light (Nikon Eclipse E400, Tokyo, Japan) and acroplan lens of 10× magnification. One image from each group was then selected for analysis.

**Figure 3:**
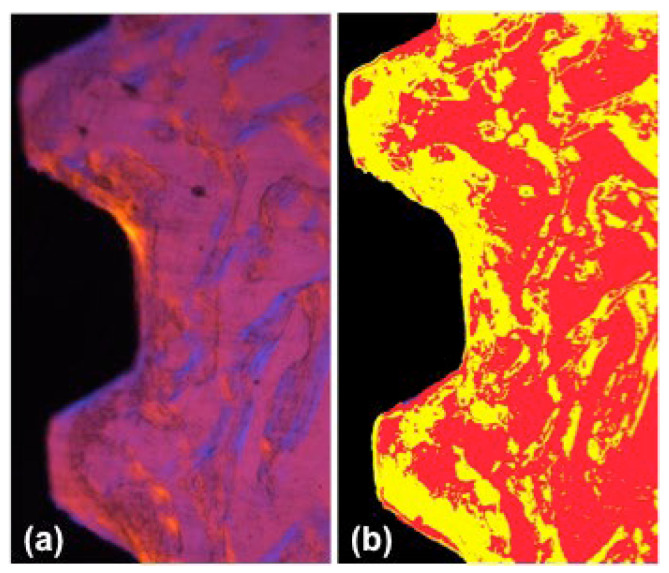
Image segmentation of the original picture. (**a**) Polarized image of the interface area; (**b**) Image segmentation with Image ProPlus, showing the implant (black), connective tissue (red), and newly formed bone (yellow). Magnification: 10×.

We standardized two screws below the cortical region in order to evaluate the newly formed bone around the implants. Image segmentation was performed using the Im-age-Pro Plus^®^ (Media Cybernetics, Silver Spring, MD, USA, v.4.5.0.29), targeted to the implant (black), connective tissue (red), and newly formed bone (yellow), as shown in Figure 3. After segmentation, the highlighted areas were counted and transported to the software Excel 2021 (Microsoft Office^®^, Redmond, WA, USA) to calculate the area/segment/image percentage. Data were analyzed using GraphPad InStat^®^ v.3.01 (San Diego, CA, USA) and using the Kruskal-Wallis test with Dunn post-test (*p* < 0.05).

## 3. Results

The SEM images of the micrometers surface roughness (Figure 2) showed no change after the HA nanometer coating, indicating a homogeneous and continuous layer over the implant surface, even though FTIR and XRD showed both the presence of OH bands and diffraction peaks, respectively, as characteristics of a well-crystallized HA.

**Figure 4:**
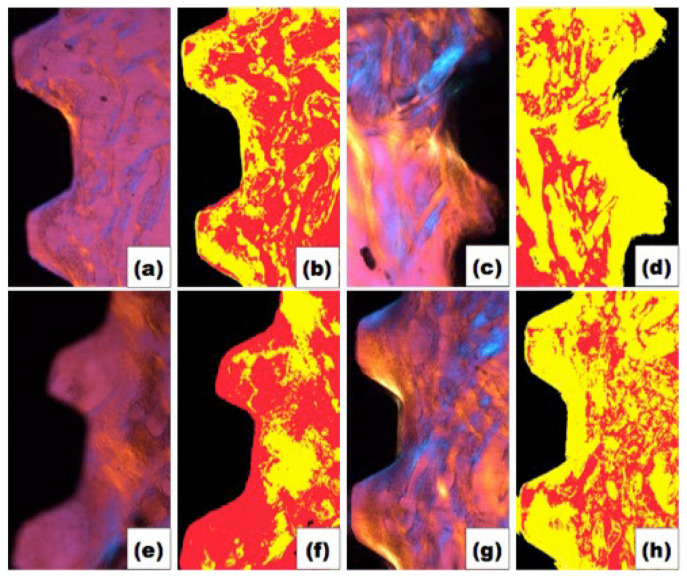
Original and segmented images of each group. (**a**,**b**) Regular instrumentation without rhBMP-7; (**c**,**d**) Regular instrumentation with rhBMP-7; (**e**,**f**) Over-instrumentation without rhBMP-7; (**g**,**h**) Over-instrumentation with rhBMP-7. Colors: BLACK—implant; RED—connective tissue; YELLOW—new bone formation.

**Figure 5:**
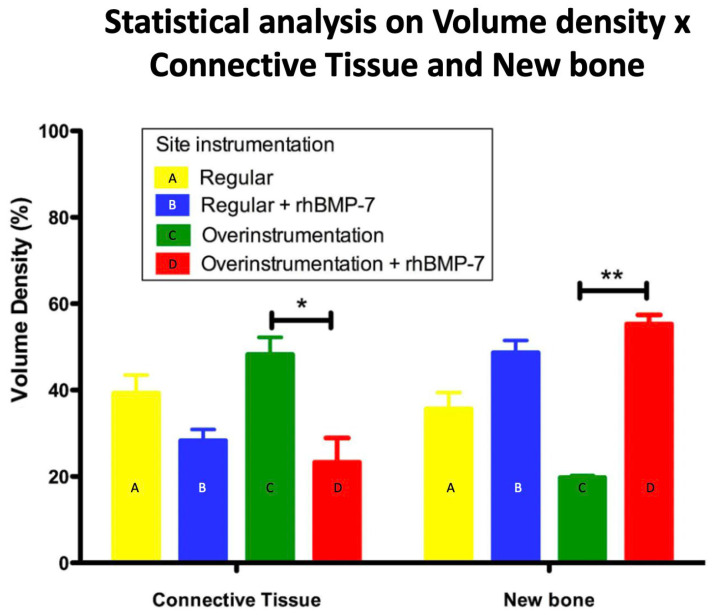
Statistical analysis of volume density of connective tissue and newly formed bone around implants with standard instrumentation and over-instrumented, with and without the addition of rhBMP-7. (* *p* < 0.05; ** *p* < 0.01).

Figure 4 shows polarized (a, c, e and g) and segmented images (b, d, f and h) per group. It is possible to verify a larger quantity of newly formed bone in the rhBMP-7 groups. In order to prove this fact, the data were analyzed (Figure 5), confirming that new bone formation was greater in groups with rhBMP-7 (b and d) compared to the average obtained by the groups without rhBMP-7 (a and c), respectively, a = 35.6%, b = 48.6%; c = 19.7%; d = 55.3%. Observing the newly formed bone, group d (over-instrumented group) was the only one that presented significant bone formation compared only to group c (*p* < 0.01). On the other hand, the area of connective tissue was smaller in groups b and d (a = 39.3%, b = 28.3%, c = 48.23%, d = 23.3%), but no significant difference was observed between groups b and d (*p* > 0.05).

## 4. Discussion

The implant biomaterial’s ability to allow hard and soft tissue healing around the implant device is strongly related to the clinical success of implant dentistry [30]. Low bone density, atrophic alveolar ridges, or immediate loading protocol constantly challenges success ratios above 90% reported for dental implantology [31]. Thus, improvements in the biomechanical systems through the implant design can cause an increase in biocompatibility and osteoconductivity or osteoinductivity, leading to faster and greater bone healing or achieving a desirable turnover [2,5,14]. In the present study, it was possible to confirm the initial positive hypothesis, in which higher volume bone density was observed for the thin-coated implants associated with rhBMP-7, significantly around the implants placed in the over-instrumented implant sites. Similar results were documented by Nemcakova et al. (2022) [28], who concluded that the BMP-7 promoted faster osseointegration and better bone healing, suggesting a possibility of earlier loading. Furthermore, Schierano et al. (2021) [32] concluded that rhBMP-7 stimulated the osteogenetic and anti-inflammatory properties when surface coated implants were used.

This work used implants coated with a thin film of HA, coated through the RAMS (Right Angle Magnetron Sputtering) system, which allowed to produce stoichiometric HA at ambient temperature. Previous studies showed the highest proliferation of human osteoblasts was achieved on HA RAMS-coated titanium substrates [19,20]. The use of sputtering coatings has been shown to eliminate some of the problems associated with the plasma-spray process, thereby increasing bone strength and the initial rate of osseointegration implants coated with HA and CaP [33]. The deposition of HA as a cover by plasma spray technique or electrochemically increased mechanical fixation and bone growth but showed no statistically significant difference between the individual applications of HA. Moreover, the addition of collagen to the mineralized phase of the coating to produce a more bone-natural surface did not increase the osteoconductive effect of HA [34].

The XRD of the Ti plates and FTIR of the dental implant analysis performed after the coating showed a thin HA film on the implant surfaces. SEM images showed no change in the implant surface morphology after the HA coating. These findings confirmed that the implants used in this work were a thin film of HA-coated implants.

In recent years, studies in vitro and in vivo experiments have been conducted to verify the effectiveness of surface treatments of implants, associating properties to improve cellular activity [19], healing and apposition bone [35,36,37]. Bone cells are known to be sensitive to the implant material’s morphology, resulting in cell bodies with distinct shapes, orientations, and adhesion processes [38,39]. Osteoblasts did not spread completely in Ti implants with rough surfaces and acquired a polygonal morphology. Moreover, the Ti implants decreased the rate of cell proliferation in early incubation [40].

Among the surface modification for orthopedic and dental implants, the addition of material based on calcium phosphate (CaP) for coating, a modification known as biomimetics, has received significant attention [14,33,41,42] due to the similarity to the essential components of natural bone. When integrated into the material’s structure, the molecules undergo a gradual release in proportion to the degradation of the layers, thus increasing the potential delivery system for osteogenic agents to the implant site [43].

Different surfaces were tested, including porous titanium oxide implants, and no significant differences were seen in osseointegration [44,45]. Conversely, elegant studies developed by Lee et al. (2010) [17] and Susin et al. (2010) [46] used rhBMP-2 and rhBMP-7, respectively, coating porous titanium oxide implants, obtained satisfactory results in the increased the alveolar ridge and also affecting resident bone remodeling. The rhBMP-2 also has great potential to increase alveolar bone, loading the implant osseointegration, and long-term function. This type of BMP increases alveolar bone integration and the predictability of clinical protocol used, changing the current treatment paradigms [47].

The combination of growth factors and titanium implants with HA-coatings shows bone growth in all dimensions of the implant when associated with ng/rhBMP-2 (non-glycosylated rhBMP-2), but without statistical differences between groups in terms of bone height and BIC, observing a higher bone density when there was no binding of HA, indicating that HA coatings may not be necessary when using non-glycosylated BMP-2 [48]. In the present study, the rhBMP-7 mammalian heterologous expression system allows the correct post-translational process, producing a glycosylated rhBMP-7 [29].

It is worth mentioning that the rhBMP-7 presented a very high osteoinduction capability since only 15min of contact with coated implants were enough to significantly increase the bone area. This handling time was significantly less than previously reported [16,46,49], suggesting an adequate working time for the potential clinical uses. A previous study testing different BMP concentrations showed that the formation of new trabecular bone was found only with 380 μg of BMP-2 [50]. Studies using porous titanium oxide implants soaked (soak-load) at high concentrations of rhBMP-2 (3.0 mg/mL) showed no increased bone formation compared to groups receiving lower concentrations (0.75 and 1.5 mg/mL) at 8 weeks [51]. Despite the present work, we could not quantify the adsorbed concentration of rhBMP-7; the concentration was less than 2.0 mg/mL. 

Stenport et al. (2003) [52] showed that 10 mg of BMP-7 associated with collagen as a carrier did not contribute significantly to the increased in titanium implant anchorage in bone. On the other hand, the association of rhBMP-7 (1.5 and 3.0 mg/mL) to the implants improved bone formation, extending above the platform of the implant after 8 weeks. The surface of the porous titanium oxide acted as an effective surface carrier for rhBMP-7, indicating a potentially clinically important stimulatory effect of local bone formation, resulting in a vertical gain of the alveolar ridge [46,49]. However, it was impossible to compare the differences in such experimental models due to the lack of a proper control group without rhBMP-7. The need for higher BMP concentrations may be due to the loss of part of the protein by the action of proteases released during the early inflammatory phase following the implant placement.

In the present work, we showed that coating HA implants with rhBMP-7 can significantly enhance new bone formation. These results agree with previous studies that used the same protein but with different implant surfaces and are not over-investigated [28,32,46]. Thus, it appears the newly formed bone observed was attributable to the protein osteoinductive effects and not to the size of the implant site, which justifies the lack of statistical significance between groups (for newly formed bone). In fact, the protein associated with the over-instrumentation accelerated local bone formation. This practice could be suitable for immediate loading protocols since we showed increased (3-fold) bone formation around implants in over-instrumented sites when compared to the control group.

A limiting factor involved in this study is related to the control of the inflammatory level since increased interleukins (pro-inflammatory cytokines) concentration also occur, which might impair the action of BMPs. Moreover, even though a better result was obtained with rhBMP-7, mainly in sites with over-instrumentation, this osteoinductive biomaterial is very expensive and may not be used routinely. Consequently, this fact reduces interest in applying it on a large scale. Therefore, we suggest that controlled clinical trials in patients with atrophic bone volume or critical-size defects may be adopted to compare the results in a clinical setting.

## 5. Conclusions

It was possible to conclude that the association of rhBMP-7 to thin HA-coated implants promoted greater new bone area than the same implants without rhBMP-7, mainly in cases of over-instrumented implant sites.

## Abbreviations

BMP	Bone morphogenetic protein
BIC	Bone-to-implant contact
Calcium and Phosphate	Ca- and P-
°C	Celsius degree
eV	electron Volts
FTIR	Fourier Transform Infrared Spectrophotometry
FTIRM-ATR	Fourier Transformed Infrared Attenuated Total Reflectance Microscopy
GIXRD	Grazing-incidence X-ray diffraction technique
HA	Hydroxyapatite
OH	Hydroxide
MPa	MegaPascal
nm	Nanometer
RAMS	Right-angle magnetron sputtering
rhBMP-7	Recombinant human bone morphogenetic protein-7
SEM	Scanning electron microscope
XRD	X-ray diffraction
